# Circadian clock during plant development

**DOI:** 10.1007/s10265-017-0991-8

**Published:** 2017-11-13

**Authors:** Keisuke Inoue, Takashi Araki, Motomu Endo

**Affiliations:** Graduate School of Biostudies, Kyoto University, Kyoto, 606-8502 Japan

**Keywords:** Circadian clock, Development, Local adaptation, Output

## Abstract

Plants have endogenous biological clocks that allow organisms to anticipate and prepare for daily and seasonal environmental changes and increase their fitness in changing environments. The circadian clock in plants, as in animals and insects, mainly consists of multiple interlocking transcriptional/translational feedback loops. The circadian clock can be entrained by environmental cues such as light, temperature and nutrient status to synchronize internal biological rhythms with surrounding environments. Output pathways link the circadian oscillator to various physiological, developmental, and reproductive processes for adjusting the timing of these biological processes to an appropriate time of day or a suitable season. Recent genomic studies have demonstrated that polymorphism in circadian clock genes may contribute to local adaptations over a wide range of latitudes in many plant species. In the present review, we summarize the circadian regulation of biological processes throughout the life cycle of plants, and describe the contribution of the circadian clock to local adaptation.

## Introduction

Plants as sessile organisms must precisely perceive environmental cues such as light and temperature to adapt their growth and development to surrounding environments. Circadian clocks are endogenous time-keeping mechanisms that allow organisms to anticipate and prepare for daily and seasonal changes in surrounding environments. Plants adjust the timing of various physiological, developmental, and reproductive processes to a proper time of day or an appropriate season based on the day length measured by the circadian clock (McClung 2006). Indeed, it has been reported that the circadian clock in plants achieves higher survival advantage and fitness (Dodd et al. 2005; Green et al. 2002). The circadian clock in plants mainly consists of interlocked transcriptional/translational feedback loops similar to the clocks in mammals or insects (Harmer et al. 2001). Previous studies have identified the core circadian clock genes, including *CIRCADIAN CLOCK ASSOCIATED 1* (*CCA1*), *PSEUDO RESPONSE REGULATOR* (*PRR*) family genes, *TIMING OF CAB EXPRESSION 1* (*TOC1*), *GIGANTEA* (*GI*), *EARLY FLOWERING* (*ELF*) genes, and *LUX ARRHYTHMO* (*LUX*), which form multiple interlocked negative feedback loops (Nohales and Kay 2016). In addition, positive regulators such as *REVEILLE* (*RVE*) genes, *LIGHT-REGULATED WD* (*LWD*) genes, and *NIGHT LIGHT-INDUCIBLE AND CLOCK-REGULATED* (*LNK*) genes have also been identified (Rawat et al. 2011; Rugnone et al. 2013; Wu et al. 2016).

**Fig. 1:**
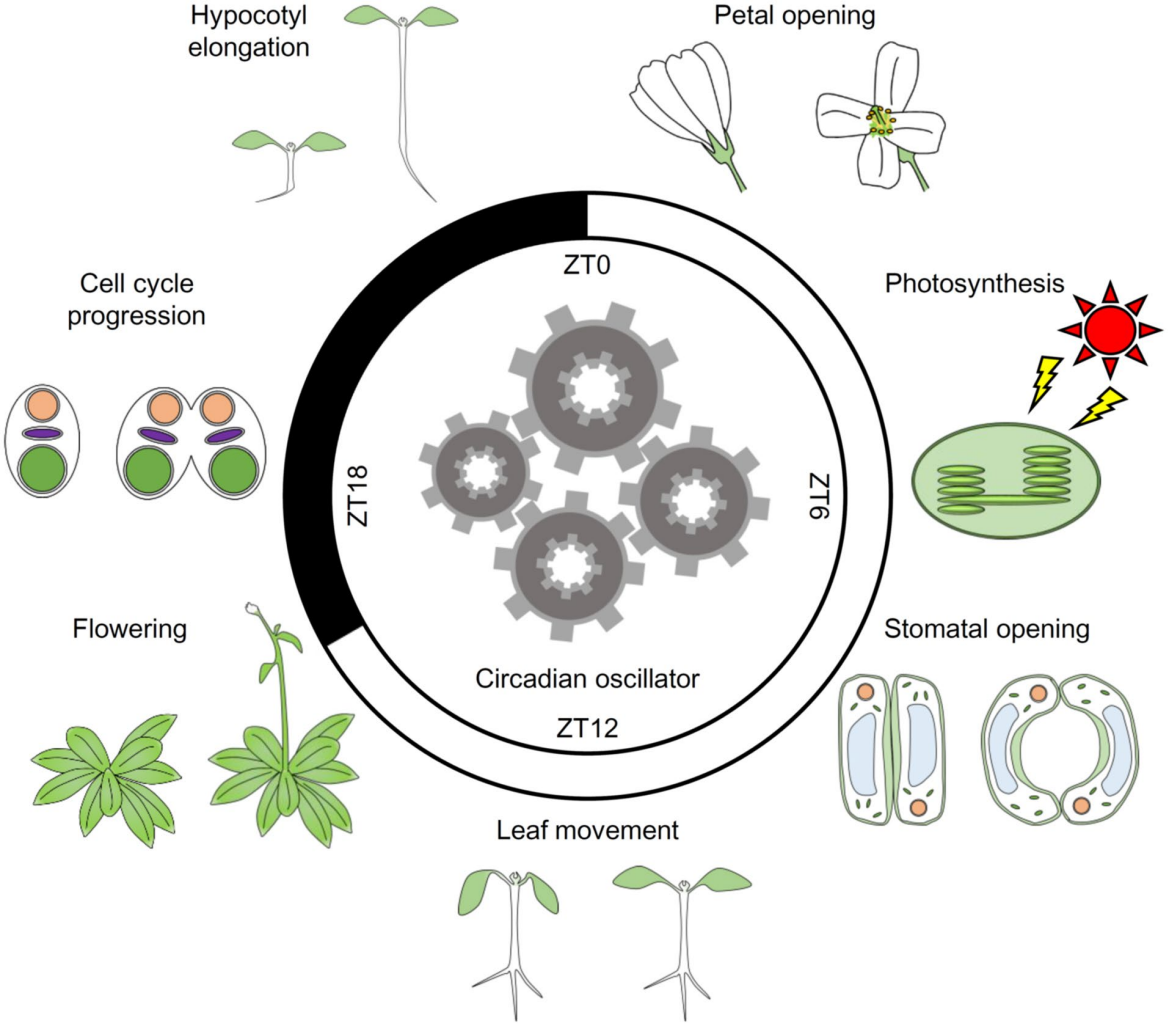
The circadian clock in plants regulates various biological processes throughout the life cycle. The circadian clock adjusts the timing of output responses, such as petal opening, photosynthesis, stomatal opening, leaf movement, flowering, cell cycle progression, and hypocotyl elongation to an appropriate time of day

The circadian clock in plants can be entrained by environmental cues, such as light, temperature, and nutrient status through multiple input pathways (Inoue et al. 2017). Then, the clock regulates various biological processes at an appropriate time of day through output pathways. Recent studies using natural accessions have revealed that altering circadian timing due to natural variation in circadian clock genes could contribute to the adaptation to local environments over a wide range of latitudes. In this review, we focus on output responses regulated by the circadian clock (Fig. 1). We summarize the circadian regulation of biological processes throughout the life cycle of plants at the cellular, tissue/organ, and individual levels.

## The role of the circadian clock at the cellular level processes

The circadian clock system is found not only in multicellular organisms but also in unicellular organisms. It has been reported that cell cycle of the cyanobacterium *Synechococcus elongatus* exhibits circadian gating, and therefore is probably regulated by the circadian clock (Mori et al. 1996; Yang et al. 2010). A time-lapse imaging revealed that phosphorylation state of the oscillator protein KaiC that is associated with elevated ATPase activity applies a circadian checkpoint on the cell division in *S. elongatus* by inhibiting FtsZ ring formation (Dong et al. 2010). Intriguingly, circadian rhythms are sustained in cells that divide three or more times during one circadian period, suggesting that cell division cycling does not interfere with the circadian clock of *S. elongatus* (Kondo et al. 1997). Clock-regulated cell cycle progression is also found in the unicellular red algae *Cyanidioschyzon merolae*. The cell cycle of *C. merolae* is highly synchronized with external light/dark cycle in which cell division occurred during the dark period (Imoto et al. 2011; Suzuki et al. 1994). The phosphorylation of the E2F protein, a key regulator for G1/S transition, is regulated by the circadian clock, resulting in the restriction of the G1/S transition to early subjective night. Furthermore, the uncoupling of cell cycle progression from circadian rhythms decreases the growth rate probably due to high oxidative stress, suggesting that the restriction of cell cycle progression during the night might be important to unicellular photosynthetic eukaryotes (Miyagishima et al. 2014). Given that the temporal circadian gating of cell division has been observed in a wide variety of eukaryotic lineages from unicellular organisms to mammals (Johnson 2010), the cell cycle of eukaryotes is closely associated with the circadian clock during the course of evolution. Although the circadian clock-coupled cell division is reported in many organisms, there is no solid evidence that the circadian clock in plants regulates cell cycle progression. However, a recent report demonstrated that the cell size of mesophyll cells in the leaf is altered in circadian clock mutants. The size of *rve 4 6 8* triple and *rve 3 4 5 6 8* quintuple mutants is increased compared with that of the wild-type plants at both seedling and adult stages of development. The average area of mesophyll cells in the leaves of *rve4 6 8* triple mutants is approximately 1.3-fold larger than that in the wild-type plants, suggesting that the increased size in the *rve* mutants is primarily due to the larger average cell size (Gray et al. 2017). Given that cell size is closely associated with the length of cell cycle (Jones et al. 2017), it is possible that the circadian clock in plants could regulate cell cycle progression as well as that in other species.

Another circadian regulation of cellular level processes is observed in stomatal opening and photosynthesis. In *Arabidopsis thaliana* (Arabidopsis) and the bean *Phaseolus vulgaris*, stomatal conductance is higher during the day than at night (Hennessey et al. 1993; Somers et al. 1998), whereas the stomata in some crassulacean acid metabolism plants *Hoya* are more open at night than during the day (Thimann et al. 1992). Additionally, photosynthesis is also regulated by the circadian clock (Dodd et al. 2014b). The expression of a large set of genes involved in the light-harvesting complex is regulated by the circadian clock (Harmer et al. 2000; Schaffer et al. 2001). Consistent with the rhythmic expression of mRNA, the accumulation level of the chlorophyll a/b binding protein (CAB) and ribulose 1,5-bisphosphate carboxylase/oxygenase activase (RCA) proteins in the tomato *Lycopersicon esculentum* Mill is under the control of the circadian clock (Martinocatt and Ort 1992; Pilgrim and Mcclung 1993). Furthermore, phosphorylation of the D1 photosystem II reaction center protein in the duckweed *Spirodela oligorrhiza* and phosphoenolpyruvate carboxylase in crassulacean acid metabolism plants is also regulated by the circadian clock (Booij-James et al. 2002; Borland et al. 1999; Hartwell et al. 1999; Nimmo 1998, 2000), indicating that photosynthesis is strictly regulated by the circadian clock at the transcriptional, post-transcriptional, and post-translational levels. In addition to photosynthesis, photorespiration, sugar metabolism, and starch degradation are also regulated by the circadian clock (Harmer et al. 2000; Lu et al. 2005; McClung et al. 2000). It has been suggested that circadian control of photosynthesis and physiology provides higher fitness for plants (Dodd et al. 2005). However, recent reports demonstrate that the circadian control of photosynthesis is not sufficient to explain the effects of the circadian clock on plant fitness. Instead, circadian control of starch degradation during the night appears to be important (Dodd et al. 2014a; Graf et al. 2010; Graf and Smith 2011), although how the circadian clock controls the rate of starch degradation during the night remains unknown.

## The role of the circadian clock at the tissue/organ level during plant development

The circadian clock in plants regulates various developmental processes throughout the life cycle of plants. At the earliest stage of plant development, the circadian clock regulates seed germination. The photoperiodic control of seed germination has been reported in many plant species (Baskin and Baskin 1976; Black and Wareing 1954, 1955; Densmore 1997), suggesting the existence of functional circadian system in seeds at least in some plant species. Indeed, imbibition, but not release from stratification can reset the circadian clock and synchronize the clocks among population of seedlings (Zhong et al. 1998). Furthermore, the circadian control of gas exchange is observed in free-running condition in dry onion seeds (Bryant 1972), suggesting that the functional circadian system is present even in quiescent seeds before germination. More recent reports demonstrated that the circadian clock controls seed germination probably through regulating a series of abscisic acid- and gibberellin-related genes expression (Covington et al. 2008; Penfield and Hall 2009).

The circadian clock in plants affects many aspects of plant growth. Hypocotyl elongation is a well-characterized growth regulated by the circadian clock. The circadian regulation of rhythmic elongation is observed in constant light (Dowson-Day and Millar 1999), although hypocotyl elongation is arrhythmic in constant dark (Nozue et al. 2007), suggesting that light signal is essential for the circadian regulation of hypocotyl elongation. This rhythmic growth is dependent on the function of two basic helix-loop-helix transcription factors, PHYTOCHROME-INTERACTING FACTOR 4 (PIF4) and PIF5. PIF4 and PIF5 act as a signaling hub to integrate various external and internal cues, such as light, clock, temperature, phytohormone, and sucrose signaling (de Lucas et al. 2008; de Montaigu et al. 2010; Feng et al. 2008; Leivar and Monte 2014; Stewart et al. 2011). It has been demonstrated that expression levels of *PIF4* and *PIF5* are well correlated with hypocotyl growth rate (Nozue et al. 2007). ELF3, ELF4 and LUX tripartite complex represses the expression of *PIF4* and *PIF5* in the early evening (Nusinow et al. 2011). In addition, ELF3 physically interacts with PIF4 to inhibit its transcriptional activity in the early night (Nieto et al. 2015). Furthermore, PIF4 and PIF5 are rapidly degraded through the interaction with light-activated PHYTOCHROME B (PHYB) upon the irradiation of light (Lorrain et al. 2008). Thus, the activity of PIF4 and PIF5 is restricted to the late night, resulting in the photoperiodic regulation of hypocotyl elongation before dawn under diurnal conditions (Niwa et al. 2009). A similar circadian regulation of elongation has been reported in other plant species such as the tomato *L. esculentum* and the red goosefoot *Chenopodium rubrum* (Fernandez and Wagner 1994; Lecharny and Wagner 1984; Tukey and Ketellapper 1963). Another circadian regulation of growth is observed in diel leaf movements. In legumes including *Mimosa pudica*, leaf movements occurred by expansion and contraction of specialized cells at the base of the petiole called the pulvinus that allows for rapid reversible changes in leaf position (Uehlein and Kaldenhoff 2008; Whippo and Hangarter 2009). Plants without pulvini, however, also undergo circadian leaf movements that at least partially depend on antiphasic differential growth of the abaxial and adaxial sides of the leaf blades and petioles (Polko et al. 2012; Rauf et al. 2013; Uehlein and Kaldenhoff 2008). Such circadian regulation of leaf movements have been observed in several plant species, such as Arabidopsis, *Brassica oleracea, B. rapa*, tobacco, and potato (Engelmann et al. 1992; Lou et al. 2011; Salathia et al. 2007; Siefritz et al. 2004; Yanovsky et al. 2000). It has been recently reported that ELF3 is required to maintain the proper phase of leaf growth and movements, although PIF4 and PIF5 are not essential to sustain rhythmic leaf growth (Dornbusch et al. 2014). These findings suggest that molecular mechanisms underlying rhythmic hypocotyl and leaf growth are different from each other. In addition, the rate of circumnutations and shade avoidance response is also under the control of the circadian clock (Niinuma et al. 2005; Salter et al. 2003; Stolarz 2009; Takase et al. 2013; Whippo and Hangarter 2009). Recently, heliotropism, solar tracking movements, in the sunflower *Helianthus annuus* is driven by antiphasic patterns of elongation on the east and west sides of the stem regulated through the coordinate action of light-signaling pathways and the circadian clock (Atamian et al. 2016).

Photoperiodic flowering is the most characterized developmental event regulated by the circadian clock (Endo et al. 2016; Shim et al. 2017). CONSTANS (CO), a key transcription factor for photoperiodic flowering, is strictly regulated by the circadian clock and light signaling pathways (Putterill et al. 1995; Samach et al. 2000; Suarez-Lopez et al. 2001; Valverde et al. 2004). CYCLING DOF FACTORs (CDFs), whose expression pattern is under the control of the circadian clock repress *CO* transcription in the morning (Imaizumi et al. 2005). Only under long-day conditions, CDFs are degraded by a complex of GI and FLAVIN-BINDING KELCH REPEAT F-BOX 1 (FKF1). The peak times of *GI* and *FKF* expression differ under short-day conditions, whereas the peaks of both expressions coincide in late afternoon under long-day conditions. Furthermore, blue-light activates FKF1 and stabilizes the GI-FKF1 complex by enhancing the interaction of GI with FKF1 before dusk. These internal and external coincidence allows the GI-FKF1 complex to target CDFs for proteasomal degradation only in the late afternoon of long-days (Sawa et al. 2007), thereby derepressing *CO* (Song et al. 2012). The stability of CO protein is regulated through light signaling pathways mediated by multiple photoreceptors. CO protein is degraded by a complex of CONSTITUTIVE PHOTOMORPHOGENIC 1 (COP1) and SUPPRESSOR OF PHYA-105 1 (SPA1) during the dark (Jang et al. 2008; Laubinger et al. 2006). In addition, CO protein is destabilized by phyB through two distinct mechanisms mediated by HIGH EXPRESSION OF OSMOTICALLY RESPONSIVE GENE 1 (HOS1) and PHYTOCHROME-DEPENDENT LATE-FLOWERING (PHL) in the morning (Endo et al. 2013; Lazaro et al. 2015), whereas phyA and CRYPTOCHROME 2 (CRY2) stabilize CO protein in the late afternoon probably by inhibiting the function of the COP1-SPA complex (Sheerin et al. 2015; Zuo et al. 2011). This coordinated action of the circadian clock and light signaling pathways allows for accumulation of CO protein in the late afternoon under long-day conditions, and then CO induces the transcription of a florigen-encoding *FLOWERING LOCUS T* (*FT*) for photoperiodic flowering (Tiwari et al. 2010). Consistent with previous findings that phyA, CRY2, COP1, SPA1, and CO all function in the phloem companion cells for photoperiodic flowering (An et al. 2004; Endo et al. 2007; Jang et al. 2008; Kirchenbauer et al. 2016; Ranjan et al. 2011), recent reports demonstrated that the circadian clock in the phloem companion cells, not in the mesophyll, epidermis, and shoot apex cells, is critical for photoperiodic flowering, suggesting the significance of vasculature-specific clock function for photoperiodic flowering (Endo et al. 2014; Shimizu et al. 2015).

At the late stage of plant development after flowering, the circadian clock regulates flower opening for successful pollination. The circadian clock restricts the timing of petal opening to a part of the day when potential pollinators are most active. In Arabidopsis, flower opens in the morning and closes at midday (van Doorn and Kamdee 2014; van Doorn and van Meeteren 2003), whereas flower of night-blooming *Cestrum nocturnum* opens in the evening and closes at dawn (Overland 1960; van Doorn and Kamdee 2014). Furthermore, some volatile compounds and nectar for attraction of pollinators are also regulated by the circadian clock, and therefore they are emitted in the correct timing of potential pollinator activities during the day (Kolosova et al. 2001; Pesti 1976; Verdonk et al. 2003). The rhythms of these compounds are likely generated through the circadian regulation of their biosynthetic genes expression (Fenske et al. 2015; Fenske and Imaizumi 2016).

## The role of the circadian clock for adaptation to local environments at the individual level

The circadian clock regulates various cellular or developmental processes and provides higher fitness under diurnal conditions as described above. Many plant species including Arabidopsis spread into different climatic and latitudinal areas with a wide range of day-length changes throughout the year. Several reports have suggested that mutations in the clock genes could contribute to adaptation to local environments. The period length of leaf movements in 150 Arabidopsis accessions is positively correlated with the day length at the latitude of origin, implying the adaptive significance of the circadian clock (Michael et al. 2003). Quantitative trait loci (QTL) analysis revealed that multiple loci interact to determine the period length, phase, and amplitude of leaf movements. *PRR7* was located at some of the QTL for circadian period, and mutants defective in *PRR* family genes exhibit altered circadian period, suggesting that polymorphism in the *PRR* genes is a candidate for adaptation to local environments. Positive correlation between the period length of leaf movements and the day length at the latitude of origin is also reported in *Mimulus guttatus* and *Glycine max* (Greenham et al. 2017). Similarly, the period length of core clock gene expression in *Capsella bursa-pastris* ecotypes is strongly correlated with the latitudinal origin (Slotte et al. 2007). *C. bursa-pastris* ecotypes derived from lower latitudes showed earlier flowering, indicating that the phase advance in clock gene expression could contribute to early flowering. Polymorphism in *ELF3* originated in Central Asia causes a short-period under light and severely dampened oscillation in the dark because of the defects in nuclear localization of ELF3, suggesting the contribution of mutations in *ELF3* to local adaptation (Anwer et al. 2014).

## Perspectives

The circadian clock regulates various cellular and developmental processes throughout the life cycle of plants, and indeed other organisms. Although accumulating evidence indicates the significance of the circadian clock for plant fitness, how the circadian system is able to regulate so many output processes and contribute to higher fitness is still largely unknown. However, recent advances of chromatin immunoprecipitation combined with massively parallel sequencing (ChIP-seq) achieved genome-wide identification of the direct regulations of clock-output genes by some of the core clock components (Ezer et al. 2017; Kamioka et al. 2016; Liu et al. 2016; Nagel et al. 2015; Nakamichi et al. 2012). Furthermore, recent analyses revealed that plants have decentralized oscillator networks consisting of multiple tissue-specific clocks, which show asymmetric couplings (Endo et al. 2014; Shimizu et al. 2015; Takahashi et al. 2015). Therefore, it is important to know how oscillator networks integrate spatial and temporal information into the whole body to regulate various responses.

As described above, polymorphism in the circadian clock genes may contribute to the adaptation to local environments. Indeed, many domesticated crops contain mutations in the core clock genes, resulting in the optimized flowering time (Nakamichi 2015). Since the day length for a given latitude is invariable in spite of the global climate changes, optimization of flowering time will continue to be needed for further crop domestication. Although modification of the circadian clock genes may enhance crop growth and in turn yields, circadian transcriptome data of crop species is still not readily available. Moreover, recent modeling of transcriptome data in field conditions versus in a growth chamber revealed that the progression of internal time in the morning and evening is different in the field and chamber probably due to the difference in increasing and decreasing rate of light intensity and temperature (Matsuzaki et al. 2015), suggesting the importance of integrating the parameters derived from controlled growth chamber into field conditions. Time-course sequences of transcriptome data of crop species in field conditions and mathematical modeling could help us to determine new targets for improving crop yields and provide new insights into the role of the circadian clock in fluctuating environments for local adaptation.
