# Correction to: Circadian clock during plant development

**DOI:** 10.1007/s10265-018-1015-z

**Published:** 2018-02-21

**Authors:** Keisuke Inoue, Takashi Araki, Motomu Endo

**Affiliations:** Graduate School of Biostudies, Kyoto University, Kyoto, 606-8502 Japan

## Correction to: Journal of Plant Research (2018) 131:59–66 10.1007/s10265-017-0991-8

The article” Circadian clock during plant development”, written by” Keisuke Inoue, Takashi Araki, Motomu Endo”, was originally published Online First without open access. After publication in volume131, issue 1, page 59–66 the Botanical Society of Japan decided to opt for Open Choice and to make the article an open access publication. Therefore, the copyright of the article has been changed to © The Author(s) 2018 and the article is forthwith distributed under the terms of the Creative Commons Attribution 4.0 International License (http://creativecommons.org/licenses/by/4.0/), which permits use, duplication, adaptation, distribution and reproduction in any medium or format, as long as you give appropriate credit to the original author(s) and the source, provide a link to the Creative Commons license, and indicate if changes were made.

